# Accelerated hyperfractionated radiochemotherapy with temozolomide is equivalent to normofractionated radiochemotherapy in a retrospective analysis of patients with glioblastoma

**DOI:** 10.1186/s13014-019-1427-5

**Published:** 2019-12-12

**Authors:** Victor Lewitzki, Rainer J. Klement, Rebekka Kosmala, Dominik Lisowski, Michael Flentje, Bülent Polat

**Affiliations:** 1 Department of Radiation Oncology, University of Würzburg, Josef-Schneider-Str. 11, 97080 Würzburg, Germany; 2 Department of Radiotherapy and Radiation Oncology, Leopoldina Hospital Schweinfurt, 97422 Schweinfurt, Germany

**Keywords:** Brain cancer, Glioblastoma, High grade glioma, Radiotherapy, Temozolomide, Corticosteroids

## Abstract

**Background:**

Current standard of treatment for newly diagnosed patients with glioblastoma (GBM) is surgical resection with adjuvant normofractionated radiotherapy (NFRT) combined with temozolomide (TMZ) chemotherapy. Hyperfractionated accelerated radiotherapy (HFRT) which was known as an option from randomized controlled trials before the temozolomide era has not been compared to the standard therapy in a randomized setting combined with TMZ.

**Methods:**

Data of 152 patients with newly diagnosed GBM treated from 10/2004 until 7/2018 at a single tertiary care institution were extracted from a clinical database and retrospectively analyzed. Thirty-eight patients treated with NFRT of 60 Gy in 30 fractions (34 with simultaneous and 2 with sequential TMZ) were compared to 114 patients treated with HFRT of 54.0 Gy in 30 fraction of 1.8 Gy twice daily (109 with simultaneous and 3 with sequential TMZ). The association between treatment protocol and other variables with overall survival (OS) was assessed using univariable and multivariable Cox regression analysis; the latter was performed using variables selected by the LASSO method.

**Results:**

Median overall survival (OS) was 20.3 month for the entire cohort. For patients treated with NFRT median OS was 24.4 months compared to 18.5 months in patients treated with HFRT (*p* = 0.131). In univariable regression analysis the use of dexamethasone during radiotherapy had a significant negative impact on OS in both patient groups, HR 2.21 (95% CI 1.47–3.31, *p* = 0.0001). In multivariable analysis adjusted for O6-methylguanine-DNA methyl-transferase (MGMT) promotor methylation status, salvage treatment and secondary GBM, the use of dexamethasone was still a negative prognostic factor, HR 1.95 (95% CI 1.21–3.13, *p* = 0.006). Positive MGMT-methylation status and salvage treatment were highly significant positive prognostic factors. There was no strong association between treatment protocol and OS (*p* = 0.504).

**Conclusions:**

Our retrospective analysis supports the hypothesis of equivalence between HFRT and the standard protocol of treatment for GBM. For those patients who are willing to obtain the benefit of shortening the course of radiochemotherapy, HFRT may be an alternative with comparable efficacy although it was not yet tested in a large prospective randomized study against the current standard. The positive influence of salvage therapy and negative impact of concomitant use of corticosteroids should be addressed in future prospective trials. To confirm our results, we plan to perform a pooled analysis with other tertiary clinics in order to achieve better statistical reliability.

## Introduction

With a proportion of 47.7%, glioblastoma (GBM) is the most common malignant tumor of the central nervous system in the USA [1]. In Germany GBM accounts for approximately 69% of all malignant brain tumors in adults [2]. Median overall survival (OS) is usually less than 15 months and 5-year OS is less than 10%.

There are several well-known prognostic factors for patients with GBM. Good validated and widely used patient-related prognostic factors include age at diagnosis, clinical and neurological performance at diagnosis, recurrence [3–6], or less common body mass index [7–9] and blood glucose levels [10–13]. Treatment related factors include complete resection, concomitant use of temozolomide (TMZ), tumor-treating fields and aggressive salvage therapy (if possible) [14–18]. Molecular tumor related factors include methylation status of the O6-methylguanine-DNA methyltransferase (MGMT) promoter [19] and mutation status of isocitrate dehydrogenase 1 and 2 (IDH 1/2) which are highly specific for secondary GBM [20–22].

A dose-effect relationship in radiotherapy of malignant gliomas was found due to the meticulous analysis of treatment data of patients treated in three Brain Tumor Study Group protocols between 1966 and 1975 [23]. Rationales for accelerated hyperfractionated therapy were postulated according to the four R’s of radiobiology: redistribution, reoxygenation, repopulation and difference in recovery from DNA damage in normal and tumor cells [24, 25]. Especially the well-known effect of accelerated repopulation of tumor cells can be partially compensated by the acceleration of treatment below the lag time of about 21 days [26]. The difference in recovery from sublethal DNA damage assuming low α/β for normal brain tissue and high α/β for tumor cells would also allow for a therapeutic gain through hyperfractionation [27].

Several randomized trials with hyperfractionated accelerated radiotherapy (HFRT) were able to deliver a proof of principle [28–31]. HFRT with or without dose escalation failed to show any superiority in overall survival (OS) or progression free survival (PFS) in comparison to normofractionated protocols [32]. Also the most recently published trial comparing dose escalated HFRT with normofractionated radiotherapy (NFRT) didn’t indicate any benefit in terms of survival in favor of HFRT [33]. The role of hypofractionated accelerated protocols was assessed in several randomized trials in mostly elderly patients [34–37].

The positive influence of adding TMZ to the accelerated course of radiotherapy was proven in randomized trials in elderly patients [34]. In a previous analysis of concomitant TMZ with hyperfractionated radiochemotherapy performed in our institution there was a survival advantage and good tolerance of simultaneous radiochemotherapy [38]. Also Kaul et al. reported good tolerance of simultaneous TMZ and HFRT [39].

Despite the abovementioned lack of benefit in terms of survival, HFRT can halve treatment time. This is the rationale to use it as an alternative scheme for patients who are willing to shorten the time of irradiation from 6 to 3 weeks.

## Materials and methods

### Patients

We retrospectively analyzed the data of 229 patients with histologically proven GBM treated at our radiation oncology department between 10/2004 and 7/2018. Treatment and survival information of patients who received NFRT with single dose of 2.0 Gy once daily to a total dose of 60.0 Gy and of those who received HFRT with single dose of 1.8 Gy twice daily to a total dose of 54.0 Gy was extracted and further analyzed. All but 4 patients received simultaneous and/or sequential chemotherapy with oral TMZ. 120 patients received chemotherapy according to the protocol of Stupp et al. beginning with radiotherapy and continuing with 6 further cycles [18]. All treatment decisions had been discussed in our institutional tumor board. Patients could choose between one of the two treatment protocols according to the anticipation of equal treatment effect and toxicity with NFRT being clearly declared as the current standard. After excluding 77 patients who had received hypofractionated treatment with or without TMZ, there remained 152 patients eligible for analysis. Of these 114 chose HFRT and 38 NFRT as their preferred treatment modality.

### Treatment planning and delivery

Radiochemotherapy was usually initiated 3 weeks after surgery. The preoperative magnetic resonance imaging (MRI), planning computer-assisted tomography (CT) with intravenous contrasting and a dedicated planning MRI with isometric voxel multiplanar reconstruction, contrast enhanced T1 and T2 fluid-attenuated inversion recovery (FLAIR) sequences were all fused to generate the planning target volume (PTV). First a clinical target volume (CTV) was defined including the resection cavity and the contrast enhanced residual or gross tumor region with a mandatory margin of 20 mm as well as any peritumoral edema. After internal validation of the influence of mask fixation on daily set up errors, a margin of 3 mm was considered appropriate to extend the CTV to the PTV. Five patients in NFRT and 22 patients in HFRT group received an additional boost up to 64 Gy to the gross tumor volume at the decision of the treating radiooncologist and internal plan discussion. If a substantial part of the brain stem (e.g. > 1 ccm) and/or chiasma was inside the PTV, modification to meet the dose constraint of 54.0 Gy for NFRT and 50.4 Gy for HFRT was performed. Radiation was delivered using highly conformal 3D-RT, intensity-modulated radiation therapy (IMRT) or − if appropriate − volumetric arc therapy (VMAT) with non-coplanar field arrangement. Data on PTV was available for 133 of 152 patients.

Chemotherapy with TMZ at 75 mg/m^2^ body surface area was initiated at day 1 of radiotherapy and was delivered daily during the whole course of irradiation. A prophylaxis against *Pneumocystis jirovecii* pneumonia was obligatory. Six cycles of adjuvant TMZ beginning at 150 mg/m^2^ for 5 days every four weeks were applied ambulatory at the neurooncology outpatient clinic starting 4 weeks after completion of radiotherapy with dose escalation to 200 mg/m^2^ for the following cycles as long as there were no hematologic toxic effects (concomitant + sequential TMZ). Four patients didn’t receive chemotherapy: three octogenarians without MGMT hypermethylation and one patient with rapid neurological impairment after the first fraction and following best supportive care. 23 Patients with hematologic toxicity, neurologic impairment or consent withdrawal during or after radiotherapy didn’t receive further TMZ (concomitant TMZ). Five patients started with TMZ after radiotherapy: three octogenarians, one patient without MGMT hypermethylation and one pregnant patient (sequential TMZ).

Follow-up was also performed by the treating neurooncologist. Clinical examination and MRI were performed every 3 months. Each case of suspected progression was discussed in a multidisciplinary tumor board with obligatory presence of a neuroradiologist, neuropathologist, neurosurgeon, oncologist and radiation oncologist.

### Statistics

The threshold for statistical significance was set at *p* = 0.005 [40]. This was to increase the credibility of our findings; in fact, from a Bayesian perspective, larger *p*-value thresholds such as the typical 0.05 value are associated with only weak evidence against the null hypothesis and may increase the risk of false positive claims [40]. OS was calculated from the day of surgery using Kaplan-Meier statistics. The log-rank test was used to determine the statistical significance of the OS difference between the treatment groups.

Fisher’s exact test and the Wicoxon rank sum test were used to determine the significance of differences between relevant prognostic factors between the treatment groups (Table 1). Univariable and multivariable Cox proportional hazard regression was performed to determine the association between variables and risk of death. To utilize as many cases as possible for multivariable modeling [41], missing covariates were imputed with multiple imputation by chained equations using the R package ‘mice’ [42]. A “missing at random” mechanism was assumed being responsible for missing variables, with all variables given in Table 1 as well as follow-up time and OS being added into the imputation model. Variables were imputed in the order of their number of missing cases. Predictive mean matching, logistic regression and a multinomial logit model were used for imputing continuous, binary and multicategorical variables, respectively. A total of 100 imputation data sets were created. Each was used to fit a Cox regression model with LASSO variable selection constraining the maximum number of variable to five in order to avoid overfitting. All variables having been selected into at least 50 out of the 100 Cox models by the LASSO method were defined as “signal variables”. The signal variables plus the treatment variable (NFRT/HFRT) were used to build a separate Cox model for each of the 100 imputed datasets, and the final Cox model was obtained by pooling the coefficients of these 100 Cox models together. For sensitivity analysis, we also built a Cox model using only the complete cases (with no missing values). All statistical analyses were performed using R version 3.5.0 and independently controlled with IBM SPSS Statistics 24.

**Table 1:** Patient characteristics, tumor and treatment related factors. Differences between the HFRT and NFRT group were evaluated using Fisher’s exact test and the Wilcoxon rank sum test for categorical and continuous variables, respectively. Unknown values were excluded in these tests. KPS: Karnofsky performance status

		Overall cohort		NFRT		HFRT		*p*-value
		(*n* = 152)		(*n* = 38)		(*n* = 114)		
Age	Median, range	59	11–81	57.5	28–75	60	11–81	0.553
Gender	Female	60	39.5%	15	39.5%	45	39.5%	1
	Male	92	60.5%	23	60.5%	69	60.5%	
Surgery extent	Complete resection	50	32.9%	8	21.1%	42	36.8%	0.0921
	Subtotal resection	52	34.2%	16	42.1%	36	31.6%	
	Debulking	11	7.2%	5	13.2%	6	5.3%	
	Biopsy	37	24.3%	7	18.4%	30	26.3%	
	Unknown	2	1.3%	2	5.3%	0	0%	
Mental status	Normal	140	92.1%	34	89.5%	106	93.0%	0.496
	Impaired	12	7.9%	4	10.5%	8	7.0%	
Neurological function	Normal	109	71.7%	29	76.3%	80	70.2%	0.537
	Impaired	43	28.3%	9	23.7%	34	29.8%	
MGMT-methylation	Yes	47	30.9%	12	31.6%	35	30.7%	0.803
	No	42	27.6%	9	23.7%	33	28.9%	
	Unknown	63	41.5%	17	44.7%	46	40.4%	
IDH-mutation	Yes	6	3.9%	1	2.6%	5	4.4%	1
	No	71	46.7%	17	44.7%	54	47.4%	
	Unknown	75	48.7%	20	52.6%	55	48.2%	
Secondary GBM	Yes	12	7.9%	4	10.5%	8	7%	0.486
	No	132	86.8%	31	81.6%	101	88.6%	
	Unknown	8	5.3%	3	7.9%	5	4.4%	
PTV [ccm]	≤269 ccm	66	43.4%	15	39.5%	51	44.7%	1
	> 269 ccm	65	42.8%	15	39.5%	50	43.9%	
	Unknown	21	13.8%	8	21.0%	13	11.4%	
Temozolomide	No	4	2.6%	2	5.3%	2	1.8%	0.364
	Concomitant	23	15.1%	4	10.5%	19	16.7%	
	Conc+sequen	120	78.9%	30	78.9%	90	78.9%	
	Sequential	5	3.3%	2	5.3%	3	2.6%	
Concomitant steroids	No	56	36.8%	19	50.0%	37	32.5%	0.0714
	Yes	77	50.7%	15	39.5%	62	54.4%	
	Unknown	19	12.5%	4	10.5%	15	13.2%	
Radiotherapy completed	Yes	145	95.4%	37	97.4%	108	94.7%	0.681
	No	7	4.6%	1	2.6%	6	5.3%	
Additional Boost	Yes	27	17.8%	5	13.2%	22	19.3%	0.470
	No	125	82.2%	33	86.8%	92	80.7%	
Salvage	No	55	36.2	8	21.1%	47	41.2%	0.0337
	Yes	68	44.7%	22	57.9%	46	40.4%	
	Unknown	29	19.1%	8	21.1%	21	18.4%	
KPS at RT start	< 70	18	11.8%	1	2.6%	17	14.9%	0.119
	70–80	64	42.1%	18	47.4%	46	40.4%	
	> 80	70	46.1%	19	50.0%	51	44.7%	
Treatment period	2004–2008	5	3.3%	1	2.6%	4	3.5%	0.779
	2009–2013	67	44.1%	19	50.0%	48	42.1%	
	2014–2018	80	52.6%	18	47.4%	62	54.3%	
In- /outpatient	Inpatient	14	9.2%	2	5.3%	12	10.5%	0.126
	Outpatient	135	88.8%	34	89.5%	101	88.6%	
	Unknown	3	2%	2	5.3%	1	0.9%	
Tumor treating fields	No	138	90.8%	35	92.1%	103	90.4%	1
	Yes	14	9.2%	3	7.9%	11	9.6%	

## Results

### Patient characteristics

From the clinical database including treatment data of 229 patients with GBM, data of 152 patients were extracted according to the treatment modality NFRT (38 patients) or HFRT (114 patients) (Table 1). Most characteristics were well matched. However, there were some more pronounced differences in the percentage of patients receiving concomitant steroids and salvage treatment. The NFRT and HFRT groups differed somewhat (*p* = 0.0921) in the percentage of complete and subtotal resection (residual tumor clearly seen or suspected on postoperative MRI). After combining complete with subtotal resection into one class and debulking with biopsy into another, the difference between the treatment groups was much less prominent (*p* = 0.556). We also used the nomogram published by Gittleman et al. [43] to calculate the individual survival prognosis for each patient to check the comparability of both cohorts. The average predicted 6-, 12- and 24-month survival probabilities for the NFRT and HFRT groups were 83% vs. 82, 52% vs. 51 and 24% vs. 23%, which was not significantly different between both groups. In this calculation, in case of unknown MGMT methylation status we always selected not methylated (accepting a negative bias for tumor that in reality were MGMT hypermethylated). MGMT-methylation status was unknown in 17 patients (44.7%) from the NFRT and 46 patients (40.4%) from the HFRT group, but the overall MGMT distribution pattern was not significantly different between both groups (*p* = 0.803).

### Survival analysis

**Fig. 1:**
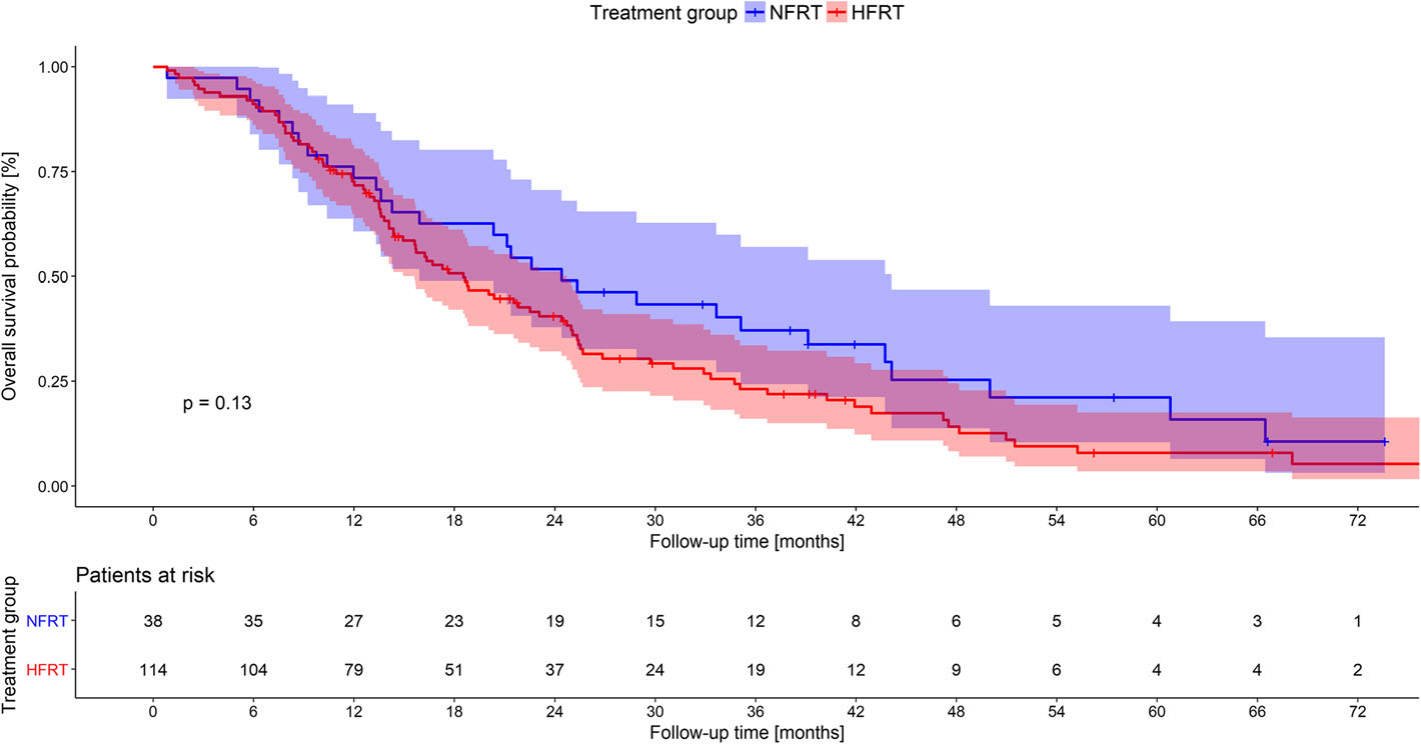
Kaplan-Meier plot of overall survival of HFRT and NFRT cohorts including 95% confidence intervals

Follow-up rate for the entire cohort was 99.34%. Out of the 152 patients, 121 (79.6%) had died at the time of analysis. Median OS was 20.3 months (95% CI 15.9–25.0) for all patients. The difference between median OS in the NFRT group (24.4 months) and HFRT group (18.5 months) (Fig. 1) was not statistically significant (*p* = 0.131).

### Regression analysis of prognostic factors

Univariable analysis revealed MGMT-methylation status, surgery extent, salvage treatment, use of steroids and TMZ as significant predictors of survival (Table 2). PTV volume (more or less than the median of 269 ccm) and age almost reached the threshold of statistical significance. Presence of IDH mutations was associated with a lower risk of death (HR = 0.167) but not statistically significant (*p* = 0.0766). In the multivariable analysis performed on the imputed datasets, the LASSO method selected MGMT-methylation status, salvage treatment and steroid use into 100 and secondary GBM into 97 out of 100 models. Inserting these signal variables together with the treatment group (NFRT/HFRT) into a new Cox model resulted in the HRs and 95% confidence intervals given in Table 3. While the treatment group was not significantly associated with OS (*p* = 0.504), there was a significant association between MGMT-methylation status, salvage treatment as well as secondary GBM with OS and steroid use almost reached the significance threshold. Using the same variables on the smaller dataset with missing variables removed resulted in similar effect estimates for all variables except for secondary GBM, which was no longer associated with risk of death. MGMT methylation and salvage treatment remained highly significant predictors of OS even with the smaller dataset.

**Table 2:** Results of the univariable Cox regression analysis. For variables with missing values, only known values were considered for model building

Variable	HR	95% CI		*p*
		lower	higher	
RT-protocol NFRT vs HFRT	1.381	0.906	2.106	0.133
Age (continuous)	1.023	1.007	1.039	0.0052
Gender: Female vs. Male	0.700	0.481	1.019	0.062
Surgery extent:				
Subtotal vs. complete resection	1.253	0.797	1.969	0.329
Debulking vs. complete resection	1.152	0.557	2.385	0.703
Biopsy vs. complete resection	2.813	1.752	4.516	**1.87 × 10**^**−5**^
Mental status: Impaired vs. Normal	0.809	0.421	1.554	0.524
Neurological function: Impaired vs. Normal	1.008	0.671	1.514	0.969
MGMT methylation: Yes vs. No	0.232	0.133	0.403	**2.23 × 10**^**−7**^
IDH mutation: Yes vs. No	0.167	0.023	1.211	0.0766
Secondary GBM: Yes vs. No	0.311	0.143	0.676	**0.0032**
PTV: > 269 ccm vs. ≤269 ccm	1.750	1.168	2.622	0.00668
Temozolomide: Yes vs. No	0.523	0.355	0.769	**0.000984**
Steroids: Yes vs. No	2.205	1.471	3.307	**0.00013**
Salvage: Yes vs. No	0.268	0.180	0.400	**1.24 × 10**^**−10**^
KPS:				
70–80 vs. < 70 90–100 vs. < 70	0.786 0.706	0.426 0.386	1.447 1.292	0.440 0.259
Treatment period:				
2008–2013 vs. 2004-2008	3.591	1.117	11.55	0.032
2014–2018 vs. 2004-2008	3.662	1.119	11.98	0.032
Tumor treating fields: Yes vs. No	0.836	0.386	1.809	0.649

0.005 *P* value entries are in bold

**Table 3:** Results of the multivariable Cox regression analysis. The HRs, 95% CIs and *p*-values for the imputed datasets are pooled from 100 Cox models, each one fitted to a particular imputed dataset

Variable	Imputed datasets (N = 152, 121 deaths)				Complete dataset (*N* = 57, 50 deaths)			
	HR	95% CI		p	HR	95% CI		p
		lower	higher			lower	higher	
RT-protocol NFRT vs HFRT	0.842	0.510	1.393	0.504	0.846	0.395	1.813	0.668
Salvage: Yes	0.243	0.140	0.421	**4.958 × 10**^**−7**^	0.231	0.115	0.465	**4.08 × 10**^**−5**^
MGMT methylation: Yes vs. No	0.286	0.160	0.513	**2.750 × 10**^**−5**^	0.265	0.132	0.529	**0.000169**
Secondary GBM: Yes vs. No	0.256	0.104	0.627	**0.00292**	0.959	0.210	4.385	0.957
Steroids: Yes	1.947	1.211	3.129	0.00597	1.930	0.994	3.744	0.0520

0.005 *P* value entries are in bold

## Discussion

The idea and biological rationale of an alternative fractionation scheme were developed in the early 1980s [24, 25]. The vast majority of protocols developed before the wide use of TMZ attempted to combine the known dose-effect relationship [23] with accelerated hyperfractionation to improve the tumor control probability of malignant gliomas. Despite the inconsistence of trial results, one point remains unanimously clear: accelerated hyperfractionation can achieve comparable tumor control probability with comparable toxicity in a shorter time frame than normofractionated irradiation. The low α/β-ratio of normal brain tissue, assumed to be 2–3 Gy [44–46], as well as a low effect of the time factor (repopulation) are both arguments to use hyperfractionated instead of hypofractionated treatment acceleration in patients with suspected better prognosis. Although limited to monocentric data, there is clinical evidence for these radiobiological considerations. Kaul et al. [39] published data of 129 patients with GBM treated with a similar protocol of 1.6 Gy twice daily to 59.2 Gy in the HFRT arm. There was comparable efficacy and tolerability of HFRT and NFRT. Probably due to the lower overall KPS-score in the whole cohort and eventually due to the inclusion of only patients with primary GBM, the median survival was lower compared to our data.

To estimate the equivalent dose in 2-Gy fractions (EQD2) for the HFRT-protocol used in our clinic we applied the D_prolif_ of 0.30 Gy/day as estimated by Pedicini et al. [47]. Calculation of a biologically effective dose (BED) by n × d(1 + d/α/β) without a time factor (e.g. the effect of tumor cell proliferation) would result in a BED of 63.72 Gy for the HFRT protocol used in our institution compared to 72 Gy for the NFRT schedule. Adjusting the dose for the gain of 21 days by accelerating the treatment in the HFRT-protocol would change the BED according to BED_tf_ = n × d(1 + d/α/β) − D_prolif_ × (t_HFRT_ − t_NFRT_), resulting in a dose of 70.02 Gy. Correction with the time factor is highly sensitive to the parameter D_prolif_. This time factor is heterogeneous in different tumor types. For example, for locoregional relapse in breast cancer it was calculated to be 0.6 Gy/day [48]. Despite this limitation, the existing data support its use in current and future analysis of accelerated radiotherapy protocols.

Salvage therapy was an important prognostic factor. There were many salvage strategies used in case of progression in our cohort. Most of them included surgery with or without subsequent irradiation or chemotherapy. Radiotherapy (hypofractionated or less frequent normofractionated or even radiosurgery) was the second frequent salvage modality after surgery. Overall 22 of 38 (57.9%) patients in the NFRT and 46 of 114 (40.4%) patients in the HFRT cohort had received salvage treatment which was suggestive for a difference (*p* = 0.0337). There is growing evidence that a salvage treatment provides a survival benefit in patients with GBM [15, 16, 49–51]. Our data provide further support for this hypothesis. Therefore we performed a primarily unplanned survival analysis of patients having received salvage treatment. In the NFRT group patients without salvage therapy had a median OS of 8.3 months (95% CI 4.2–12.4) compared to 10.2 months in the HFRT group (95% CI 7.0–13.4). Salvage therapy resulted in a very similar median OS of 25.3 months (95% CI 12.6–38.0) in the NFRT and 25.4 months (95%CI 18.9–31.9) in the HFRT-group. The non-significant difference between median OS in the whole NFRT group (24.4 months) and HFRT group (18.5 months) was therefore probably due to patients without exact information on salvage therapy. In those, mean OS was 55.8 months in NFRT and 41.7 months in HFRT (median OS was not reached). We can only speculate that most patients with unknown status for salvage therapy did receive salvage treatment elsewhere. Started or continued use of corticosteroids during radiochemotherapy of GBM has been shown to influence the treatment outcome negatively [52–54]. It is obvious that patients without gross tumor resection or with impaired neurological function are at higher risk of initiating or continuing treatment with corticosteroids. This was also the case in our study. In the univariable and multivariable Cox model corticosteroid administration was a strongly negative predictor with a high hazard ratio of almost 2 which is concordant to the abovementioned reports. Mechanisms by which corticosteroids could negatively interfere with radiotherapy of GBM and thus affect OS have been described by Pitter et al. [54]. One further mechanism is their well-known ability to raise blood glucose levels, which in turn could increase radioresistance of GBM cells [13]. Our confirmation of previous reports on an association between corticosteroid use and risk of death is alarming and should motivate further investigations.

### Study limitations

Due to the retrospective nature of our analysis no clear recommendation for the clinic can be derived. Twelve patients had secondary GBM with prior diagnosed anaplastic astrocytoma or oligodendroglioma. Survival analysis of these patients started with the first treatment after histological diagnosis of GBM. Despite this procedure the overall survival of this group of patients was better than in the subgroup with primarily diagnosed GBM. In eight other patients who did not receive surgery in our institution, the original histology report could not be found in our electronic database but only in clinical reports. For these patients we assigned a status of unknown considering the primary or secondary nature of GBM. In general, unknown values for a number of variables (Table 1) are a major limitation of this study, although we tried to overcome this limitation to some extent with multiple imputation and checked the consistency with an analysis on the cases for which every variable value was known.

## Conclusions

Our retrospective analysis supports the hypothesis of equivalence between HFRT and the standard protocol of treatment for GBM. For those patients who are willing to obtain the benefit of shortening the course of radiochemotherapy, HFRT may be an alternative with comparable efficacy although we point out that it was not yet tested in a large prospective randomized study against the current standard. The positive influence of salvage therapy and negative impact of concomitant use of corticosteroids should be addressed in future prospective trials. We also plan to perform a pooled analysis with other tertiary clinics in order to achieve better statistical reliability.

## Data Availability

The datasets generated during and/or analyzed during the current study are not publicly available due to data safety considerations/limitations of author’s institution.

## Abbreviations

BED: Biologically effective dose
CT: Computer-assisted tomography
CTV: Clinical target volume
DNA: Deoxyribonucleic acid
FLAIR: Fluid-attenuated inversion recovery
GBM: Glioblastoma
HFRT: Hyperfractionated accelerated radiotherapy
IDH 1/2: Isocitrate dehydrogenase 1 and 2
IMRT: Intensity-modulated radiation therapy
KPS: Karnofsky performance status
MGMT: O6-methylguanine-DNA methyl-transferase
MRI: Magnetic resonance imaging
NFRT: Normofractionated radiotherapy
OS: Overall survival
PTV: Planning target volume
TMZ: Temozolomide
VMAT: Volumetric arc therapy
